# Neurotrophic factors and hippocampal activity in PTSD

**DOI:** 10.1371/journal.pone.0197889

**Published:** 2018-05-25

**Authors:** Ümit Tural, Ahmet Tamer Aker, Emin Önder, Hatice Turan Sodan, Hatice Ünver, Gür Akansel

**Affiliations:** 1 The Nathan S. Kline Psychiatric Research Institute, Orangeburg, New York, United States of America; 2 Department of Psychiatry, Medical Faculty of Kocaeli University, Kocaeli, Turkey; 3 Department of Child and Adolescent Psychiatry, Medical Faculty of Marmara University, Istanbul, Turkey; 4 Department of Radiology, Medical Faculty of Kocaeli University, Kocaeli, Turkey; Stellenbosch University, SOUTH AFRICA

## Abstract

Although numerous studies have investigated the neurotrophic factors and hippocampal activity in posttraumatic stress disorder (PTSD) separately each other, it is unclear whether an association between neurotrophic factors and hippocampal activity is present. The aim of this study was to evaluate the functional changes in hippocampus before and after treatment with escitalopram and to associate these changes with peptides related to neuronal growth in patients with chronic PTSD and trauma survivors without PTSD. Fifteen earthquake survivors with chronic PTSD and thirteen drug naïve trauma exposed individuals without PTSD underwent fMRI scans in a block design. Serum levels of Nerve Growth Factor (NGF) and Brain Derived Neurotrophic Factor (BDNF) were measured before and after 12 weeks treatment with escitalopram. Baseline median serum level of NGF was significantly lower in patients with chronic PTSD than trauma survivors; however, 12 weeks of treatment with escitalopram significantly increased it. Higher activation was found both in left and right hippocampus for chronic PTSD group than trauma survivors. Treatment with escitalopram was significantly associated with suppression of the hyperactivation in left hippocampus in patients with chronic PTSD. Bilateral hyperactivation in hippocampus and lowered NGF may associate with neurobiological disarrangements in chronic PTSD. Treatment with escitalopram was significantly associated with both improvement in the severity of PTSD symptoms and biological alterations. Patients diagnosed with PTSD may have further and complicated deteriorations in hippocampal networks and neurotransmitter systems than individuals who had not been diagnosed with PTSD following the same traumatic experience.

## Introduction

In individuals with post traumatic stress disorder (PTSD) exposure to reminders of the trauma is associated with self-reported distress, changes in peripheral psychophysiologic measures [1–3] and increases in regional cerebral blood flow (rCBF) in certain brain areas such as anterior temporal poles, amygdala, insular cortex, orbitofrontal cortex and posterior cingulate cortex in patients with Post Traumatic Stress Disorder (PTSD) [4–9]. Advances in structural and functional neuroimaging (i.e. Blood oxygen level dependent contrast imaging-BOLD) in PTSD patients have provided information regarding neuroanatomical correlates of PTSD and have implicated the involvement of the amygdala, hippocampus, anterior cingulate, Broca’s area, medial prefrontal cortex and visual cortex [6, 8–12]. Studies have reported both structural [13] and metabolic abnormalities in hippocampus following a wide range of tasks such as recognition of neutral targets [14], continuous performance task [7], encoding of neutral word pairs [15], face—profession name pairs [16], correct recognition of trauma-related words [17] and as well as at rest [18]. Moreover, positive linear correlations between hippocampal activation and symptom severity have been reported in patients with PTSD [19].

Studies in both animals and humans have shown that stress is associated with changes in hippocampal function and structure, an effect that might be mediated through decreased neurogenesis, increased glucocorticoid levels, and/or decreased levels of BDNF [20]. BDNF is highly expressed in hippocampus, the major area of active neurogenesis in human adult brains [21, 22]. Despite the plenty of evidence on negative correlation between BDNF and stress, only a few controlled studies and a case report have reported a lower serum BDNF in subjects with PTSD than healthy subjects [23]. Angelucci et al. (2014) compared BDNF serum levels in subjects with trauma exposure who did not develop PTSD and subject developed PTSD. They found lower BDNF serum levels in patients with PTSD as compared to individuals who do not develop PTSD following traumatic experience. Controversely, other studies found that patients with PTSD had higher BDNF levels than controls [24–28]. Those levels are higher right the traumatic event and six months later [27], decreasing over time, and were not significantly different from the controls after the 1-year of traumatic experience [26]. Nerve Growth Factor (NGF) is another important member of the neurotrophins family [29]. It is remarkable that the importance of NGF for neurons in vivo was established almost immediately by demonstrating that injection of NGF antibody caused death of sympathetic neurons. NGF, like other neurotrophins, is important in communication of nerve cells, during development of the nervous system, and in neuroplasticity, memory, and learning in the adult nervous system [29]. However, the level of NGF in PTSD has not been reported yet.

It might be important to observe the changes in hippocampal activity and level of circulating neurogrowth factors associated with clinical response or improvement during the SSRI treatment for the projections to the future. Vermetten, Vythilingam, Southwick, Charney, & Bremner (2003) found that treatment of PTSD patients for a year with paroxetine resulted in a 5% increase in hippocampal volume and a 35% improvement in verbal declarative memory function [30]. Concordant with this finding, a SPECT study of anxiety disorders (including PTSD) before and after treatment with citalopram revealed a significant deactivation in the superior and anterior cingulate, right thalamus and left hippocampus [31]. It has been reported that serum BDNF predicts responses to escitalopram, which is a SSRI, in chronic PTSD [32].

The purpose of the study is to examine whether trauma survivors with and without PTSD are different in hippocampal activation, circulating NGF and BDNF, and to assess the effect of escitalopram on the mentioned variables in trauma survivors with PTSD.

## Materials & methods

### Participants

An earthquake measuring 7.4 on the Richter scale killed 18,243 people and left more than 250,000 people homeless. We reported the prevalence of PTSD among survivors as 11.7% three years after the earthquake in a previous community-based study [33]. Fifteen right-handed, treatment seeking female patients with the earthquake-related *chronic* PTSD and 13 right-handed female survivors of the earthquake survivors without PTSD, participated in the study. Only female survivors included in the study in order to avoid from confounding effect of sex. Participants with medical or neurological co-morbidity, cerebral damage or a history of substance or alcohol abuse were excluded from the study. Patients with PTSD and trauma survivors were assessed with the SCID at the baseline, and all patients entering the study were rated with the Clinician-Administered PTSD Scale (CAPS) at baseline, but only patients with chronic PTSD assessed on week 2, 4, 6, 8 and week 12.

### Study design

A repeated measures design was used. Baseline hippocampal activities and serum levels of NGF and BDNF (see below) were measured for both *chronic* PTSD patients and trauma exposed non-PTSD group. After the 12-week escitalopram trial was completed in patients with chronic PTSD, their hippocampal activities during the negative stimuli challenge test, NGF and BDNF serum levels were measured again. Patients received a fixed dose of 10 mg/day escitalopram. All previous psychotropic medications had been withdrawn for at least one week before the start of treatment with escitalopram. The present prospective study has been approved by the local ethics committee (#28-1/27), was carried out in accordance with The Declaration of Helsinki and written informed consent was obtained from all participants before inclusion in the study.

#### Clinician administered PTSD scale (CAPS)

CAPS was administered to the subjects by an experienced clinician in psychological trauma. It is used to diagnose of PTSD according to DSM IV criteria. The scale explores the prevalence and intensity of each symptom of PTSD by scoring them between 0–4 separately. Turkish version of the CAPS has been formed and found to be a reliable and valid tool for PTSD. Moreover, the Turkish version of CAPS has shown good interrater reliability and high concordance with SCID [34].

#### NGF and BDNF measurements

In order to measure NGF and BDNF plasma levels, 5 mL of blood was withdrawn from each subject by venipuncture into an anticoagulant-free vacuum tube. The blood was immediately centrifuged at 3000 × *g* for 5 min, and serum was kept frozen at -80 C until assayed. BDNF and NGF serum levels were measured using sandwich-ELISA, using a commercial kit according to the manufacturer’s instructions (Chemicon International, USA). The intra-assay and inter-assay variations were 3.7%–8.5%, and 4.1%–8.9% for BDNF and NGF respectively. The sensitivity was 15 pg/ml for BDNF and 10–15 pg/mL for NGF with an upper range of detection of 1000 pg/mL.

#### MRI technique

A 1.5 T MR unit (Gyroscan S15 Intera; Philips Medical Systems, Eindhoven, The Netherlands) was used, with a quadrature head coil. For the blood-oxygen-level dependent (BOLD) contrast study, T2* weighted axial gradient echo echoplanar images covering the cerebrum were obtained using the following parameters: TR = 3000ms, TE = 50ms, flip angle = 90°, slice thickness = 5.5 mm, slice gap = 1.2 mm, image matrix 64x64, field of view (FOV) = 23x23 cm. For anatomical reference, high resolution inversion recovery images were obtained (TR = 1400, TE = 15, TI = 350, image matrix = 384x512, prescribed with the same field of view and slice thickness-gap parameters as the BOLD images). Foam padding was fitted snugly between the subject’s head and a Plexiglas head cradle within the head coil to immobilize the subject’s head. We designated the hippocampus as an a priori region of interest (ROI) because of atypical hippocampal function and structure to be associated with trauma [10–11]. The delineation of the left and right hippocampus ROIs were based on Talarairach definitions in standart stereotactic space. The contrast of interest was the Earthquake pictures vs. rest. The hippocampus was traced on the anatomical images and then placed into Talairach space using the image processing software. Signal from the hippocampus was extracted, and the number of voxels significantly different from 0 at p≤0.001 was recorded.

#### Challenge procedure

Scanning of the negative stimulus-traumatic imagery condition using fMRI was measured at baseline and endpoint of the study. During the adjustment period, each subject was instructed to lie still, breathe through her nose for a 5 minutes in the MRI scanner and then to begin focusing on the earthquake-related traumatic images. For the challenge procedure, we used photographs of collapsed buildings due to the earthquake and projected the material via a projector on a screen placed in front of the MRI scanner. By means of an angled mirror positioned approximately 10 cm above the participant’s eyes; the screen was visible for the participant. Following an empty (white) screen for 10 sec (rest), the traumatic image was shown for an additional 10 sec (exposure). The experiment involved 16 rest periods and 16 exposureperiods; periods repeated 32 times, continued for 320 sec totally. As soon as the subject saw the image, she was encouraged to remember olfactory, auditory, somato-sensory, and visual sensations that were associated with the traumatic event. The data was analyzed using the propriety software on the workstation provided by the vendor (Philips, Eindhoven, Netherlands). The first 16 dummy scans were excluded from the analysis. Initially, the phase wrapping artifacts and the surrounding bone were masked by manually altering the default mask value of 62 to cover as much of the neural tissue as possible while excluding the calvarial structures. The following parameters were then selected: Spatial: 3x3 kernel, Temporal: None, Trend correction: None, Window: 3, Period length: 32. The hemodynamic parameters were zero to 2 and the threshold was 0.63. The activated pixels above this level were placed on the corresponding anatomical structure on a high resolution T1 weighted image and counted manually for each participant on all slices that display the target anatomical structure. The spatial extend analysis within the hippocampus was used in order to further investigate between group differences in the volume of ROI activation. All participants have subjective report to indicate that the procedure elicited significant recollection after the challenge procedure.

#### Statistics

We calculated the overall means, medians and percentages according to covariates of interest as descriptive statistics. The main outcome measures were the baseline difference and changes in the BOLD signal, NGF and BDNF serum levels after treatment. We used Shapiro—Wilk test to evaluate if data were normally distributed. Non-normally distributed variables reported as median (mdn) ± interquartile range (IR). Sociodemographic variables were assessed either with independent samples t-test or Mann-Whitney U tests for continuous variables or chi-square test. Yates’ corrected *χ*^*2*^ test was used for all other 2×2 tables. Fisher’s exact test was used instead of chi-square test when a 2×2 table had a cell with an expected frequency of less than 5. We used Mann-Whitney U tests to evaluate differences between independent groups without normal distribution. MANOVA was used in the comparison of CAPS’ subscales between the groups. Univariate ANOVAs with Bonferroni correction were used following a significant global multivariate F test. Repeated data (before and after the treatment) on continuous variables were explored by ANCOVA and ANCOVA or Wilcoxon signed rank test and Friedman test depending on distrubutional features. For nonparametric correlation analyses Spearman’s rho (*r*_*s*_) tests were used. All statistical tests were evaluated using PASW Statistics 18 at a two-sided 0.05 level of exact significance. Significance values of nonparametric tests procedures were reported as exact significance. If a significant *p* value has been yielded, *r* value was reported as the effect size estimate.

## Results

The study included 15 female patients with chronic PTSD and 13 female trauma survivors with no past or current psychiatric disorder. The mean duration of chronic PTSD was 35.9±6.8 months. Four patients had depression and 3 had panic disorder as comorbid with chronic PTSD. The charesterictics and comparisons between the groups can be seen in Table 1.

**Table 1:** Characteristics of the participants.

	Baseline Assesments			After Treatment	
	PTSD (N = 15)	Trauma Survivors (N = 13)	Statistics	PTSD (N = 15)	Statistics
Age (Mean±SD)	42.53±8.73	43.31±12.10	t = -0.20, df = 26, p = 0.846		
Previous trauma history (n, %)	8 (53.33)	9 (69.23)	χ2 = 0.22, df = 1, p = 0.638		
CAPS (Mean±SD)					
Re-experience subscale	23.33±3.73	6.92±3.75	F_(1,26)_ = 133.87, p<0.0005^a^	5.53±3.04	F_(1,14)_ = 625.34, p<0.0005^b^
Arousal subscale	20.00±3.74	2.00±1.91	F_(1,26)_ = 244.45, p<0.0005^a^	3.20±1.26	F_(1,14)_ = 314.60, p<0.0005^b^
Numbing subscale	16.13±5.48	1.77±1.24	F_(1,26)_ = 85.29, p<0.0005^a^	4.93±1.79	F_(1,14)_ = 58.75, p<0.0005^b^
Avoidance subscale	13.73±3.31	2.31±0.48	F_(1,26)_ = 151.82, p<0.0005^a^	1.87±1.25	F_(1,14)_ = 224.48, p<0.0005^b^
Total score	73.20±11.66	13.00±5.05	F_(1,26)_ = 297.14, p<0.0005^a^	15.53±4.67	F_(1,14)_ = 529.315, p<0.0005^b^
NGF (Mdn±IR pg/ml)	14.50±2.75	18.00±1.96	z = -2.654, p = 0.007	16.50±2.75	z = -2.442, p = 0.012
BDNF (Mdn±IR pg/ml)	451.89±193.79	456.00±78.06	z = -1.014, p = 0.322	490.50±106.20	z = -2.897, p = 0.002
BOLD activation at (Mdn±IR pixel)					
Left hippocampus	3.00±2.00	2.00±1.00	z = -2.308, p = 0.019	2.00±1.00	z = -1.889, p = 0.092
Right hippocampus	6.00±2.00	2.00±3.00	z = -3.677, p<0.0005	5.00±2.00	z = -2.859, p = 0.002

^a^Univariate ANOVAs following a significant global multivariate ANOVA (F_4,23_ = 79.913, Pillai’strace = 0.933, p<0.0005);

^b^Repeated Measures ANOVA following a significant global multivariate ANOVA (F_4,11_ = 203.53, Pillai’strace = 0.987, p<0.0005); CAPS, Clinician administered posttraumatic stress disorder scale; NGF, Nerve growth factor; BDNF, Brain derived neurotrophicfactor; BOLD, Blood-oxygen-level dependent.

### Neurotrophic factors

As seen in Table 1, baseline median level of NGF was significantly lower in patients with PTSD than the trauma survivors with a large effect size (*r* = 0.50). Treatment with escitalopram significantly associated with an increase in mean circulating NGF level from baseline level in patients with PTSD (Table 1; *Wilcoxon’s z* = -2.44, *p* = 0.012). The magnitude of treatment effect on NGF was medium (*r* = 0.45). Hence, there was no significant difference in circulating NGF between trauma survivors and patients with PTSD treated with 12 weeks of escitalopram (Table 1; *U* = 75.0, *z* = -1.04, *p* = 0.316). Neither median BDNF blood levels between trauma survivors with PTSD and trauma survivors at baseline (Table 1) nor at the end of study (*U* = 84.0, *z* = -0.622, *p* = 0.547) were significantly different. However, the treatment with escitalopram significantly increased serum BDNF level from baseline (Table 1) in the PTSD group (*Wilcoxon’s z* = -2.90, *p* = 0.002, *r* = 0.53).

### Hippocampal activity

**Fig 1:**
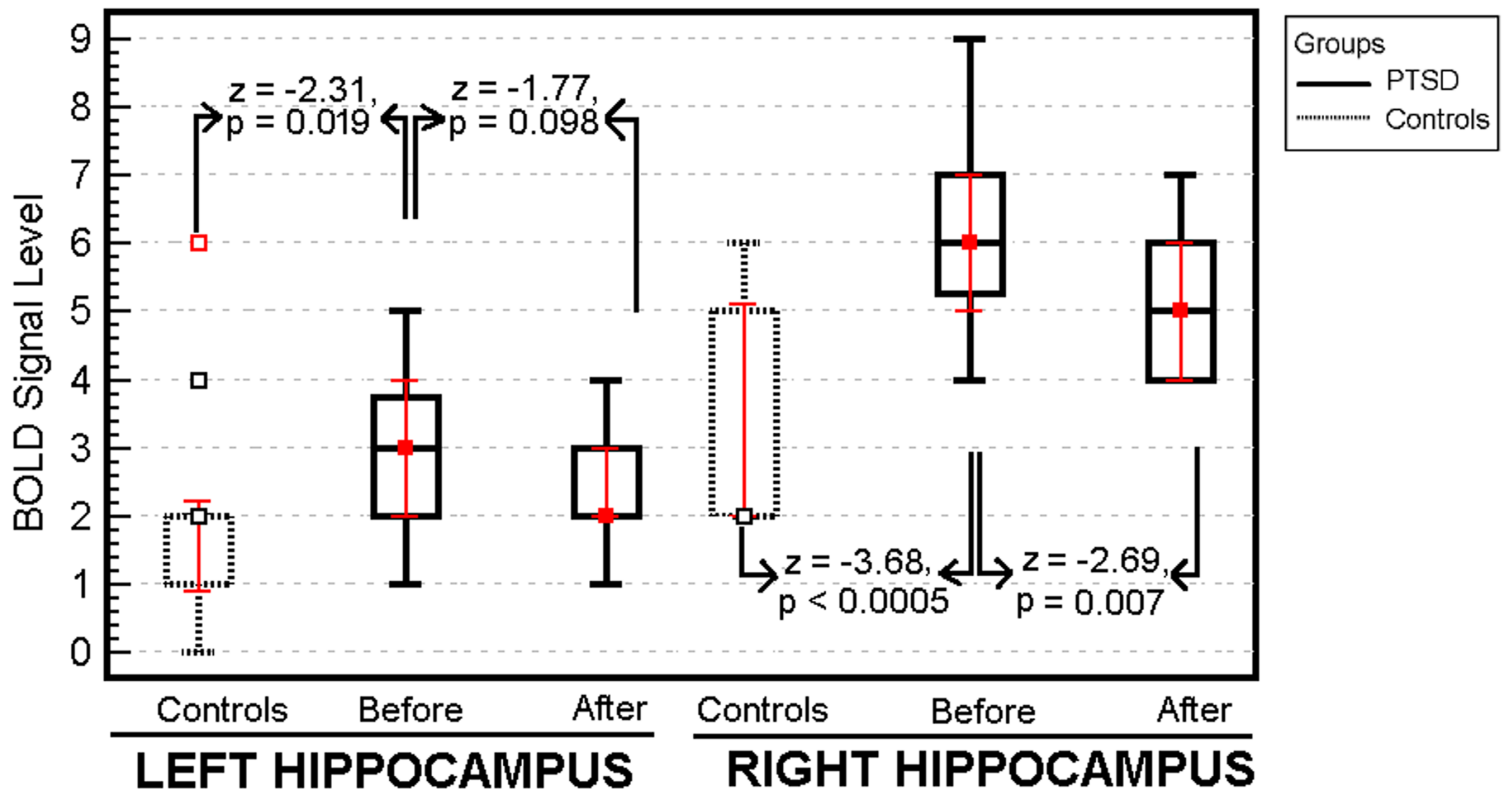
Mean BOLD signal activations in right and left hippocampus of the patients with and without PTSD following the same traumatic experience. There was a significant decrease in BOLD signal levels in the right hippocampus in patients with PTSD after treatment.

Patients with chronic PTSD showed significantly greater BOLD signal activation in both right and left hippocampus than the trauma survivors without PTSD following the traumatic imagery challenge procedure (Table 1). Attributed effect size of PTSD for differences at the baseline are *r* = 0.70 and 0.44 for right and left hippocampus respectively. There was a significant decrease in BOLD signal levels in the right hippocampus in patients with PTSD following the treatment (Wilcoxon’s z = -2.86, p = 0.002, *r* = 0.74) (Fig 1). Nevertheless, there was no significant change in the level of BOLD signal in the left hippocampus before and after treatment with escitalopram in patients with PTSD (*Wilcoxon’s z* = -1.89, *p* = 0.092).

### Clinical change

All patients completed the study and tolerated citalopram without any significant adverse event. Total Clinician-Administered PTSD Scale (CAPS) scores assessed over 3 months were significantly decreased from baseline to endpoint (73.20±11.66 to 15.53±4.67; *Freidman χ*^*2*^ = 60.84, *df* = 5, *p*<0.0005) throughout week 6 (61.40±9.24; *Wilcoxon’s z* = -3.33, *p* = 0.001, *r* = 0.61), week 8 (37.20±17.15; *Wilcoxon’s z* = -3.35, *p* = 0.001, *r* = 0.61) and week12 (15.53±4.67; *Wilcoxon’s z* = -3.41, *p* = 0.001, *r* = 0.62) in patients with chronic PTSD.

### Correlations between neurotrophic factors, hippocampal activity and clinical change

**Fig 2:**
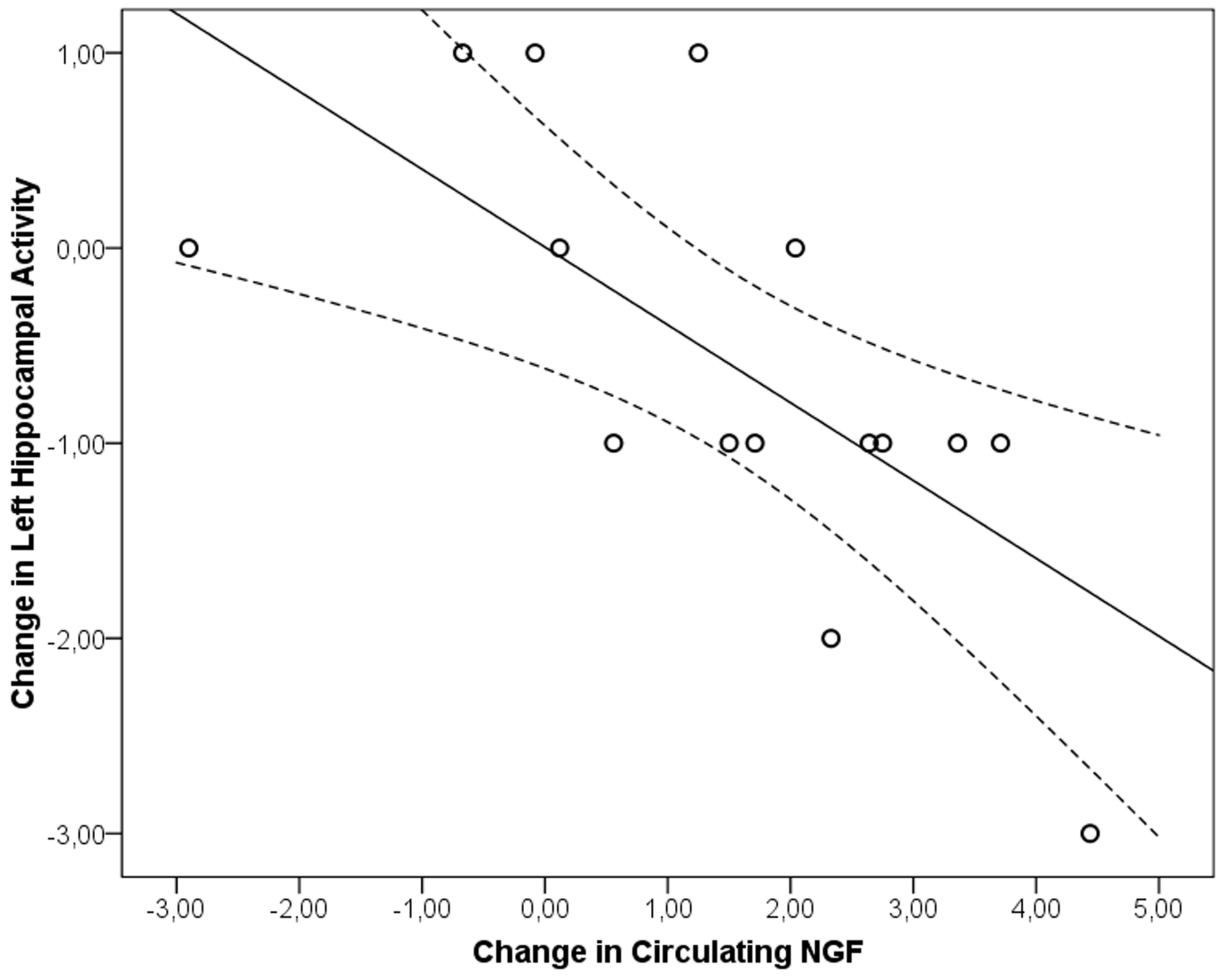
Correlation between blood-oxygen-level dependent (BOLD) signal changes in left hippocampus and circulating NGF in patients with PTSD. ^a^Linear Fit Method R^2^ = 0.454, dotted lines represent 95% CI for mean (*r*_*s*_ = 0.529, *df* = 15, *p* = 0.043).

Δ (change) CAPS scores, ΔNGF and ΔBDNF blood levels, Δright and Δleft hippocampus BOLD activation inter-correlations showed a significant negative correlation between ΔNGF and Δleft hippocampal BOLD activation level solely (*r*_*s*_ = -0.748, *df* = 15, *p* = 0.001; Fig 2). It was also found that Δ right and Δ left hippocampus BOLD activations were significantly positively correlated (*r*_*s*_ = 0.529, *df* = 15, *p* = 0.043). A repeated measures ANCOVA by including biological measurements as covariates performed on CAPS subscales revealed that ΔNGF(F_(4,7)_ = 4.790, p = 0.035), Δright (F_(4,7)_ = 5.482, p = 0.025) and Δleft hippocampus (F_(4,7)_ = 8.798, p = 0.007) BOLD signal were associated with changes in CAPS subscales. Further univariate analyses showed that changes in the avoidance subscale change was associated with both Δright (F = 11.134, df = 1, p = 0.008, r = 0.548) and Δleft hippocampus BOLD change (F = 21.553, df = 1, p = 0.001, r = 0.674). ΔNGF significantly associated with changes in avoidance (F = 11.126, df = 1, p = 0.001, r = 0.548) and numbness (F = 5.960, df = 1, p = 0.035, r = 0.432) subscales of CAPS.

## Discussion

The basic finding of the current study is increased activity in both right and left hippocampus during the challenge test in patients with PTSD in comparison to trauma exposed individuals. This is in line with some of the previous studies reporting an increased activity in hippocampus. For example, Whalley, Rugg, Smith, Dolan, & Brewin (2009) found significantly greater hippocampal responsivity during the recognition of neutral targets in PTSD compared to depressed subjects and trauma-exposed control subjects [14]. Hippocampal hyper-responsivity has also been demonstrated during plenty of challenge procedures such ascontinuous performance task [7], encoding of neutral word pairs [15], face—profession name pairs [16] and recognition of trauma-related words [17], as well as at rest [18]. Positive correlations between hippocampal activation and symptom severity have been previously reported, but, we could not replicate it [19,35]. Yet, we found that changes in NGF and BOLD signals from both hippocampi were significantly associated with changes in CAPS subscales. The remarkable finding of the present study is that the right hippocampus seems to be strongly associated with PTSD than the left. The magnitude of effects that attributable to PTSD on the increased activity in hippocampus are medium to large (*r* = 0.44 for left, *r* = 0.70 for right). However, treatment of escitalopram could not suppress the hyperactivation in left hippocampus. The difference in size of hyper activity in left and right hippocampus may be associated with PTSD itself and reflect an additional disordered cognitive and memory processes beyond that associated with trauma exposure only.

Another major finding of the study is that the patients with chronic PTSD have a lower serum level of NGF than trauma exposed non-PTSD individuals, and 12 weeks treatment of escitalopram has a positive effect in restoring NGF production in patients with PTSD. The size of effect which attributable to PTSD on NGF difference between the groups is large (*r* = 0.50) at the baseline. Effect size of the medication on NGF increase is medium (*r* = 0.45). We have found that ΔNGF have a significant negative correlation with Δleft hippocampal activation. Thus, an increase in NGF levels associated with a suppression of left hippocampus hyperactivation driven by traumatic images or vice versa. This relationship may create a new framework for future medications, which would push neuronal mechanisms to increase NGF level, in the treatment of PTSD. Both low NGF levels and hyperactivity in the right hippocampus—but not in the left hippocampus were successfully normalized after escitalopram treatment in patients with *chronic* PTSD. Similiarly, in a SPECT study, Peres et al. (2007) reported increased left hippocampal activation in subjects with subthreshold PTSD, and following psychotherapy this abnormality has been normalized [36]. Taken together, these findings strongly support the hypothesis that chronic PTSD has mediated or associated with biological alterations in hippocampus. As far as we know, there is no study in the literature exploring the relationship among NGF, PTSD and antidepressants yet. NGF is a member of the neurotrophin family, which is involved in a variety of signaling events, such as cell differentiation, proliferation and survival, growth cessation, and apoptosis of neurons [37]. There are some studies, indirectly suggesting that there might be an association between NGF, BDNF and PTSD. For example, stress, either in chronic [38] or acute forms [39] can change NGF concentration. Further, increase in NGF concentration can affect memory directly in rodents [40] or indirectly by NGF being carried to the Stellate Ganglion via a retrograde transport in rats [41]. CBT significantly increases the NGF level in patients who has a response to treatment [42]. Some authors have suggested that NGF might be the key element between memory consolidation and PTSD [43]. These findings have suggested that NGF might have mediated some of the symptoms of PTSD.

Recent meta-analyses have confirmed significantly lower BDNF protein levels for depressed patients relative to concentrations found in healthy controls [44], with levels normalizing after treatment with antidepressants [45]. Given the overlapping symptoms and high level of comorbidity between depression and anxiety disorders, and the communities in their pathophysiology, one can assume that BDNF levels in anxiety disorders may mirror the changes found in depression. Some of the studies have reported a lower serum BDNF in subjects with PTSD than healthy control subjects [26,46]. However, a meta-analysis exploring the association of BDNF with several anxiety disorders have reported no significant association of serum BDNF level with PTSD [47]. We have supported the latest by reporting no significant different serum BDNF levels in subjects with PTSD than those of trauma exposed non-PTSD group in our sample. The relationship of BDNF to pathophysiology of depression or anxiety disorders and the mechanism of drug action remains to be determined, although, it seems that escitalopram do not change serum BDNF levels [32,48]. By contrary, we have found a significant increase in serum BDNF levels with a large effect size (*r* = 0.53) which induced by escitalopram treatment in the present study.

A few preliminary open-label studies suggest that escitalopram is both efficacious and well tolerated in patients with PTSD [32,49,50]. Our findings support the previous studies by the mean global CAPS score significantly decreased from 73.20±11.66 at baseline to 15.53±4.67 at the end of the study. Besides, no one discontinued because of adverse events. Nevertheless, the readers should consider the limitations of the study such as open label design, relatively short treatment period and psychotherapy was not controlled. Like many other medications, escitalopram is off-label in PTSD.

There are limitations of this study include a relatively small number of subjects and current comorbidity in the PTSD group. However, it is well known that comorbidity is extremely common among individuals with PTSD, and whether the results of this study were influenced by the presence of comorbid disorders is unclear. We also did not measure neurocognitive or memory functions. The changes in hippocampal activity could be partly explained by practice effects. Briefly, it is unclear that which part of our findings is attributable to underpinning biology of PTSD or traumatic experience. Readers also should be aware of that this study could not determine a causal relationship between hippocampal hyperactivity, neurotrophic factors and PTSD. On the other hand, to include only females may be seen as strength of the study. Sex hormones [51,52] or age [53] may have influence on expression and activity of BDNF and NGF. Including only age matched females might be protecting the current study from confounding effect of sex on neurotrophic factors.

## Conclusions

The results were reported here are broadly consistent with previous findings of functional abnormalities in hippocampi in PTSD. We found bilaterally hyperactivity in hippocampus (but more severe in the right) in response to negative imagery stimulus in patients with PTSD. NGF has also been found lower in trauma survivors with PTSD than those of without PTSD. Twelve weeks of treatment with escitalopram was significantly associated with increased NGF levels and decreased hippocampal activity as well as improvement in CAPS scores. At least, some of the abnormalities have been normalized following the treatment. The main conclusion of the study would be that PTSD heavily damages the neuronal networks and viability in comparison to trauma exposed population without traumatic stress syndrome.

## Supporting information

S1 Database Deidentified SPSS full dataset of the study. (SAV)
